# Ultrasound and MR-imaging in preoperative evaluation of two rare cases of scar endometriosis

**DOI:** 10.1186/1757-1626-1-97

**Published:** 2008-08-18

**Authors:** George Pados, John Tympanidis, Menelaos Zafrakas, Dimitrios Athanatos, John N Bontis

**Affiliations:** 1 1st Department of Obstetrics and Gynecology, Aristotle University of Thessaloniki, Thessaloniki, Greece

## Abstract

Scar or incisional endometriosis is a rare, often misdiagnosed, pathologic condition of the abdominal wall. Two cases of incisional endometriosis are presented. Both patients presented with atypical cyclic pain and palpable nodules on scars of previous cesarean sections. In both cases, the mass was totally excised, after accurate preoperative evaluation with 2-D ultrasound, power Doppler and MRI. Microscopic examination confirmed the preoperatively presumed diagnosis of cutaneous endometriosis. In cases of suspected scar endometriosis, preoperative diagnostic imaging is valuable in determining the extent of disease, thus enhancing accurate and total excision.

## Introduction

Extraperitoneal endometriosis, i.e. the presence of ectopic, functional endometrium outside the peritoneal cavity is exceedingly rare. Cutaneous endometriosis is a form of extraperitoneal endometriosis, sometimes associated with previous laparoscopic or open abdominal operative procedures [1]. Diagnostic imaging may be used for accurate preoperative diagnosis and evaluation of the extent of cutaneous endometriosis lesions, but publication of such images in the medical literature has been scarce, due to the rarity of this condition. We present herein two cases of cutaneous endometriosis following cesarean section, both systematically evaluated preoperatively with 2-D ultrasound, power Doppler, and MRI.

## Case presentation

### Case 1

**Figure 1:**
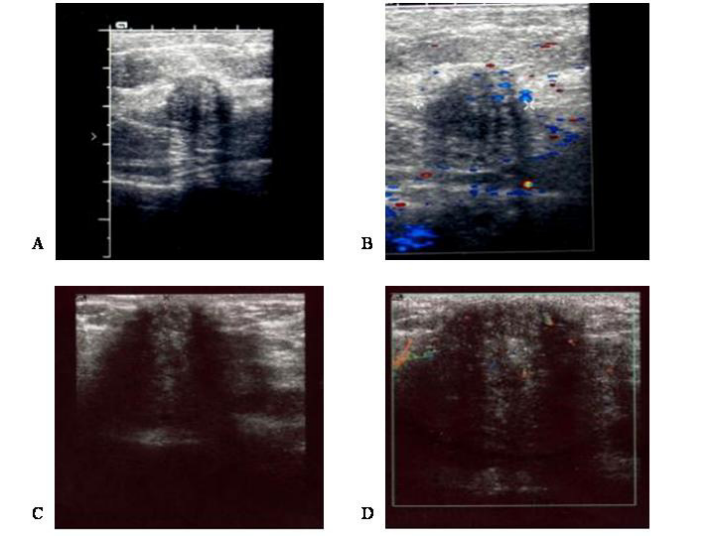
**2-D and power Doppler sonographic views of scar endometriosis nodules**. Using 7.5 MHz transducers, scar endometriotic lesions appeared hypoechoic, with internal echoes on 2-D ultrasound (A, C), and with internal vascularity on power Doppler (B, D). A and B: Case 1; C and D: Case 2.

**Figure 2:**
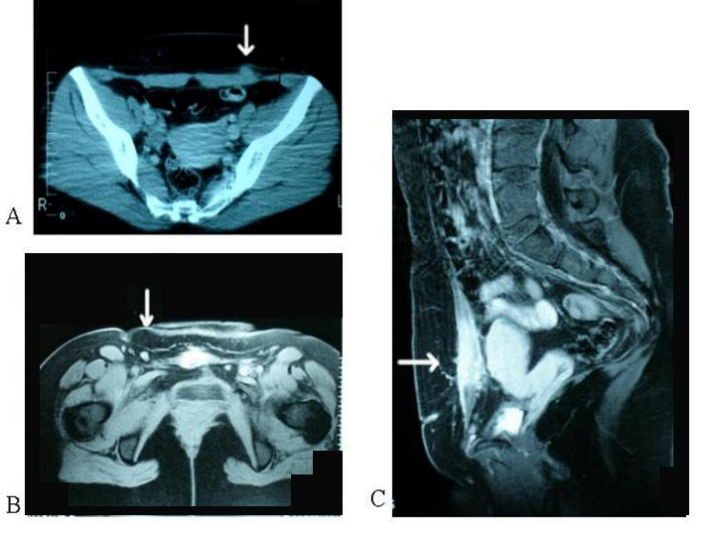
**MRI of scar endometriosis nodules**. Endometriotic lesions (arrows) appear isodense to muscle on transverse (A, B) and axial (C) T1-weighted spin-echo MRI. A: Case 1; B and C: Case 2.

A 28 year-old white woman, G2P1, presented with atypical cyclic pain on a small, firm lump at the outer margins of the scar of a previous cesarean section, performed five years earlier. Patient history details were as follows: Occupation: housewife; Ethnicity: Greek; Weight 75 Kg; Height: 165 cm; Medical history: one previous cesarean section, otherwise unremarkable; Family history: unremarkable; Patient habits and medications: non-smoker, no alcohol consumption, no current medications. Sonographic examination of the abdominal scar showed a hypoechoic mass, with internal echoes, infiltrating the subcutaneous fat, extending up to the sheath of the rectus abdominis muscle (Figure 1A). Power Doppler showed internal vascularity within the lesion (Figure 1B). MRI showed that the lesion was isodense to muscle and confirmed its localization in subcutaneous fat tissue (Figure 2A). The lesion was removed surgically and diagnosis of endometriosis was confirmed histologically.

### Case 2

A 29 year-old white woman, G2P1, presented complaining of atypical cyclic pain on the scar of a previous cesarean section, performed six months earlier. Patient history details were as follows: Occupation: Secretary; Ethnicity: Greek; Weight: 65 Kg; Height: 167 cm; Medical history: one previous cesarean section, otherwise unremarkable; Family history: unremarkable; Patient habits and medications: non-smoker, no alcohol consumption, no current medications. On clinical examination, a firm lump was found on the abdominal scar. Ultrasound scan showed a large hypoechoic mass with internal echoes, measuring approximately 10 mm, located at the lateral margin of the scar (Figure 1C). Power Doppler sonography showed increased vascularity within the mass (Figure 1D). On static MRI the mass appeared isodense to muscle (Figure 2B and 2C), while T2-weighted imaging showed increased signal-intensity within the lesion. After total surgical excision, histological examination showed that the mass consisted of endometrial tissue and stroma, suggesting cutaneous endometriosis.

## Discussion

Cutaneous endometriosis developing on scars of previous operations is a rare condition. Scar or incisional endometriosis is usually confined in superficial layers of the abdominal wall, but it may sometimes infiltrate deeper layers and present in exceptional cases even as a uterocateneous fistula [2]. Various theories have been proposed concerning the etiopathogenesis of endometriosis in general, including retrograde menstruation, metaplasia, and venous or lymphatic dissemination [3,4]. Many patients with scar endometriosis do not have any signs or prior history of peritoneal endometriosis, suggesting that this condition might be probably caused by endometrial cell dissemination into the wound at the time of surgery. Since the proliferative capacity of end-differentiated cells is limited, transfer of endometrial stem cells to incisions of the abdominal wall at the time of uterine surgery, followed by proliferation at the new site, seems to be the most plausible explanation for development of scar endometriosis.

Incisional endometriosis presents typically as a firm, palpable lump at the site of a surgical scar, usually accompanied by cyclic pain and swelling during menses. Cyclic bleeding may also occur. Cyclic symptoms and signs should alert to clinical diagnosis of endometriosis. Macroscopically, the mass is usually non-discrete, rubbery and often multiloculated, containing chocolate cysts. Differential diagnosis includes hernias, lipomas, hematomas, abscesses, cheloids, suture granulomas, sebaceous cysts, as well as malignant tumors, including desmoid tumors, sarcomas, lymphomas or primary malignancies of the skin and metastatic tumors [5].

Due to the rarity of incisional endometriosis, there is no data available concerning cost-effectiveness of different diagnostic methods. At a first glance, the simplest and less costly approach would be excisional biopsy followed by histological examination of the lesion, without prior imaging evaluation. However, this may lead to inadequate excision, and subsequently disease recurrence, necessitating re-excision. On the other hand, preoperative evaluation with imaging techniques can facilitate total surgical excision. On 2-D sonography, scar endometriosis lesions may appear as cystic or multicystic, mixed or solid masses, with internal vascularity on power Doppler. Though these findings are not specific [6], 2-D sonography allows preoperative evaluation of the extent of such lesions. If 2D- and Doppler-ultrasound studies seem inadequate, the extent and biologic behaviour can be further evaluated by MR-imaging. T1-weighted MRI shows lesions isodense to muscle, while T2-weighted images show high signal intensity with marked enhancement [7]. Thus, operative resection can be planned accurately and safely, particularly in recurrent and extensive lesions infiltrating deeper layers of the abdominal wall.

Combined oral contraceptives, progestogen-only therapy and GnRH-analogues have been used in the therapeutic management of cutaneous endometriosis, but recurrence is common after discontinuation of treatment. On the other hand, wide excision of the whole lesion, even if this necessitates fascial excision, leads to permanent cure. Recurrence is rare following surgical treatment, and is usually attributed to inadequate excision [8].

## Conclusion

In conclusion, use of diagnostic imaging, including 2-D ultrasound, power Doppler sonography and MRI, in the preoperative assessment of suspected scar endometriosis lesions is very helpful for accurate determination of the extent of disease. This approach enhances total surgical excision, which is crucial for definitive diagnosis and avoidance of disease recurrence.

## Abbreviations used

2-D: Two-dimensional; MR(I): Magnetic Resonance (Imaging); G: Gravida; P: para; GnRH: Gonadotropin Releasing Hormone.

## Competing interests

The authors declare that they have no competing interests.

## Authors' contributions

GP conceived the study and participated in patient management acquisition of data, interpretation of data, and was a major contributor in writing the manuscript. JT participated in patient management, acquisition of data, and drafting of the manuscript. MZ revised critically the manuscript adding substantial intellectual content. DA participated in patient management, acquisition of data, and drafting of the manuscript. JB coordinated the study and patient management and revised critically the manuscript. All authors have read and approved the final manuscript.

## Consent

Written informed consent was obtained from both patients – in their native language – for publication of this case report and accompanying images. Copies of the written consent are available for review by the Editor-in-Chief of this journal
